# Iatrogenic aortic valve injury following mitral valve surgery: A systematic review

**DOI:** 10.34172/jcvtr.025.33350

**Published:** 2025-09-28

**Authors:** Michele D’Alonzo, Besart Cuko, Julien Ternacle, Olivier Busuttil, Nabil Dib, Serge Sicouri, Carlo De Vincentiis, Louis Labrousse, Thomas Modine, Basel Ramlawi, Massimo Baudo

**Affiliations:** ^1^Cardiac Surgery Unit, Poliambulanza Foundation Hospital, Brescia, Italy; ^2^Department of Cardiology and Cardio-Vascular Surgery, Hopital Cardiologique de Haut-Leveque, Bordeaux University Hospital, Pessac, France; ^3^Department of Cardiac Surgery Research, Lankenau Institute for Medical Research, Main Line Health, Wynnewood, PA, USA; ^4^Department of Cardiac Surgery, IRCCS Policlinico San Donato, Milan, Italy; ^5^Department of Cardiac Surgery, Lankenau Heart Institute, Main Line Health, Wynnewood, PA, USA

**Keywords:** Iatrogenic aortic regurgitation, Mitral valve surgery, Complication, Review

## Abstract

Iatrogenic aortic regurgitation after mitral valve surgery is the consequence of either direct stitching-related perforation or indirect distortion of aortomitral fibrous continuity by the mitral ring/prosthesis. This review aims at describing the reported cases of iatrogenic aortic valve regurgitation following mitral valve surgery, focusing primarily on its management. PubMed, ScienceDirect, DOAJ, and Cochrane databases were searched from inception until December 2023 for case reports and case series describing iatrogenic aortic valve regurgitation following mitral valve surgery. The literature review found 17 articles describing 20 cases of new onset aortic valve regurgitation after mitral valve surgery. Among them, 5 patients did not undergo reoperation, either due to medical decision or patient refusal. The non-coronary cusp was predominantly affected (11 cases), the left coronary cusp involved in 4 cases, and a mixed mechanism occurred in 5 cases. Subsequent surgical interventions included aortic valve replacements in 5 cases and aortic valve repair in 4 cases. A suggested management decision algorithm is finally proposed. Iatrogenic aortic valve regurgitation after mitral valve surgery remains an unfortunate complication. Attention should be given to prevent this complication. Intraoperative transesophageal echocardiography plays a crucial role for early detection. Management strategies vary from medical therapy to surgical interventions. The reparative strategy requires a surgical procedure associated with significant mortality.

## Introduction

 Severe aortic regurgitation (AR) remains an uncommon but significant complication following mitral valve interventions. While infective endocarditis stands as a commonly recognized culprit for aortic leaflet injury,^1^ the ex-novo development of aortic valve perforation following cardiac interventions raises suspicion of an iatrogenic origin. Usually, AR is a consequence of either direct stitching-related perforation or indirect distortion of the sub aortic curtain due to sutures retaining the mitral valvular ring/prosthesis. Iatrogenic AR has also been documented in the aftermath of blunt chest trauma, percutaneous coronary intervention, Impella (Abiomed Inc., Danvers, USA) device placement, ventricular or atrial septal defect repair, and left ventricular septal myectomy.^2,3^ These injuries, including tear, perforation, or tethering of any of the three cusps, should be detected by transesophageal echocardiography (TEE) during the intervention before cardiopulmonary bypass (CPB) is weaned. When it occurs, the mechanism and severity of AR should be determined to define the optimal strategy between watchful waiting and reintervention. This review aims at describing the reported cases of iatrogenic AR following mitral valve surgery, focusing primarily on its clinical impact and management.

## Methods

 This systematic review adhered to the Preferred Reporting Items for Systematic Reviews and Meta-Analyses (PRISMA) guidelines.^4^ The complete research protocol has been registered with the PROSPERO systematic review registry under the code CRD42024526349. Because this study analyzed data from previously published sources and did not involve direct patient participation, it did not require approval from a research ethics board or patient consent. The data supporting the findings of this study can be obtained from the corresponding author upon reasonable request.

###  Literature Search

 PubMed, ScienceDirect, DOAJ, and Cochrane databases were searched from inception until December 2023 for case reports and series describing iatrogenic aortic valve regurgitation following mitral valve surgery. The search strategy is presented in Supplementary file, Table S1. In addition, the bibliography of all studies and reviews were searched to identify further articles. We included studies published in English. Abstracts, presentations, reviews, editorials, letters, and comments were excluded.

###  Study Selection

 This review included studies documenting iatrogenic aortic regurgitation after mitral valve surgery. All forms of mitral valve surgeries were included. The review considered case reports, case series, and clinical studies regardless of patient number, but excluded reviews and non-English papers. To prevent patient overlapping in cases of multiple publications from the same institution, the study period was evaluated, and the publication with the largest sample size was included.

###  Data Extraction

 Data extraction was carried out using Microsoft Office 365 Excel software (Microsoft, Redmond, Washington, USA). Whenever possible, individual patient data were collected. The data extracted included sample size, study period, study center, sex, age at intervention, main procedure, echocardiographic finding, and treatment strategy. Variations in the reported information were observed across cases, with each article providing unique variables not present in others. Denominators for reporting data were determined either explicitly or inferred based on the presence or absence of specific variables to obtain individual patient data. However, some variables might have had missing data.

 The quality of the included case reports and non-randomized clinical studies was assessed using the Joanna Briggs Institute critical appraisal tool.^5^

## Results

###  Selection of Studies

**Figure 1:**
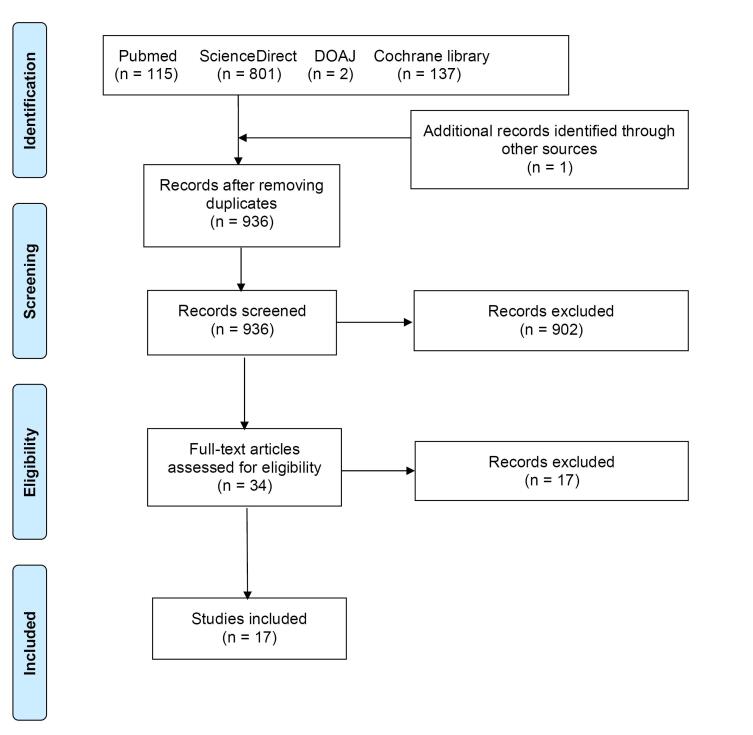
Preferred Reporting Items for Systematic Reviews and Meta-analysis (PRIMA) flow of included studies

 The systematic review flow is detailed in Figure 1. Initially, the literature search identified 1055 potentially relevant studies, with one additional article discovered through backward snowballing. After duplicate removal, 936 studies remained for screening. Out of these, 34 full-text articles were evaluated for eligibility, and 17 articles,^6-22^ encompassing a total of 20 patients, met the inclusion criteria. These studies were published between 1996 and 2022, as shown in Table 1. The critical appraisal of the included studies is provided in Supplementary file, Table S2.

**Table 1:** Outline of the included studies

**Author**	**Year**	**Study**	**Country**	**N° of Patients**	**Age**	**Main Surgery**	**Echo evaluation**	**Second Surgery**	**Outcome of second surgery**
Aboelnasr ^6^	2013	Case report	Egypt	1	32	MV repair	Perforation NCC discovered after 6 weeks	AV Repair	Discharged
Al Yamani ^7^	2012	Case report	France	1	60	MV replacement (mechanic valve)	NCC e LCC lesions discovered after 8 months	AVR, Bentall as bailout procedure for the aortic annulus rupture	Died after 36h
Alshoubi ^8^	2022	Case report	USA	1	62	MV replacement (biological valve)	Intra-op: commissure between NCC and LCC cut by mitral bioprosthesis suture	Reinforcement sutures applied	Discharged
Dogan ^9^	2013	Case report	Turkey	1	30	MV replacement (biological valve)	NCC prolapse discovered after 11 years	Not done	
Dreyfus ^10^	2011	Case report	France	1	48	CABG + MVRepair	Moderate AR after 5 years	Not done	
Ducharme ^11^	1999	Case report	Canada	1	54	CABG + MVRepair	Intra-op: aortic wall was folded by sutures	Cut and re-saw three point of anulus	Discharged
Hill ^12^	1997	Case series	USA	3	39	MV replacement (biological valve)	NCC perforation discovered after 3 years	AV repair	Discharged
					66	MV replacement (biological valve)	Intra-op: NCC perforation	AV replacement	Died after 4 years
					71	Redo of MV replacement (biological valve)	LCC perforation discovered during main hospitalization	Not done	Died for transfusion reaction
Koh ^13^	2002	Case report	England	1	50	AV replacement 12 years before, MV replacement (biological valve)	Intra-op: Teflon pledgets from the sutures used to secure the mitral valve had caused the tilting disk valve to trip	The aortic valve was, therefore, excised and rotated so that interference of the valve mechanism by the Teflon was avoided	Discharged
Kolakalapudi ^14^	2015	Review + case report	USA	1	28	MV replacement (mechanic valve)	Tethered NCC discovered during main hospitalization	Not done	Patient refused second operation, discharged
Lakew ^15^	2016	Case report	Germany	3	NA	MV repair (minimally invasive)	Intra-op: NCC perforation	AV repair	Discharged
Lee ^16^	1996	Case report	Canada	1	57	MV replacement (mechanic valve)	Distortion of the base of aortic valve discovered after 4 years	Heart Transplantation	Died after HT
Mehta ^17^	2007	Case report	USA	1	79	MV replacement (biological valve)	Intra-op: LCC perforation	AV replacement	Died on OR
Oakley ^18^	2013	Case report	USA	1	24	Redo of MV replacement (mechanic valve)	NCC perforation discovered after 2 months	AV repair	Discharged
Rother ^19^	2000	Case report	USA	1	46	MV repair	Intra-op: tethered LCC	AV replacement	Unknown
Santiago ^20^	2011	Case series	USA	2	37	CABG + MV repair	Restricted motion of Left coronary cusp discovered after 6 days	AV replacement	Discharged
					67	CABG + MV replacement (mechanic valve)	Intra-op: NCC blocked by stitch	AV replacement	Discharged
Spina^21^	2018	Case Report	USA	1	71	5° Redo MV replacement (biological valve)	CT reconstruction: a surgical pledget can be seen near the left coronary cusp and commissure	TAVI Evolut R 34	Discharged
Uygyr ^22^	2018	Case report	Turkey	1	62	MV replacement (mechanic valve)	NCC cusp lesion discovered after 12 years	Not done	

Legend: AV: aortic valve; CABG: coronary artery bypass grafting; LCC: left-coronary cusp; MV: mitral valve; NCC: non-coronary cusp

###  Outcomes

 The initial mitral valve surgeries comprised 6 mitral valve repairs and 14 mitral valve replacements, 6 with mechanical prosthetic valves and 8 with bioprosthetic valves. Three patients underwent a cardiac reoperation, and four patients a coronary artery bypass grafting concomitant procedure.

 The non-coronary cusp was predominantly affected (11 cases), the left coronary cusp was involved in 4 cases, and a mixed mechanism occurred in 5 cases. Subsequent surgical interventions included aortic valve replacements in 5 cases and aortic valve repair in 4 cases. Among the included patients, 5 patients did not undergo reoperation,^9,10,12,14,22^ due to either medical decision or patient refusal.

 Four deaths after the second surgery were reported, during either the main hospitalization or after the late discovery of iatrogenic aortic regurgitation.

## Discussion

###  Anatomical Consideration

 A profound comprehension of the intricate relationship between mitral valve surgery and aortic valve leaflet perforation requires a meticulous review of cardiac anatomy. The aortic valve, centrally positioned among the four valves, assumes a pivotal role in the cardiac skeleton. Fibrous extensions radiating from the aortic valve reach the annulus of the mitral and the tricuspid valves. The right and left fibrous trigones contribute significantly to the cardiac skeleton, establishing a direct continuity between the mitral and aortic valves. Consequently, surgical interventions on one valve invariably bring about substantial modification in the geometric dynamics of the other.^23^ Each aortic cusp, which is susceptible to be injured depending on its relative anatomical position, is exposed to different risks. In general terms, the left coronary cusp is particularly vulnerable during mitral valve surgery, the non-coronary cusp during mitral and tricuspid valve surgery, and the right coronary cusp during pulmonary valve surgery and ventricular septal defect repair, Figure S1.^12,14^

###  Clinical Impact

 AR after mitral valve surgery often leads to acute lesions that can manifest themselves even before the end of cardiac surgery, particularly during CPB weaning. The patient’s heart may encounter challenges in transitioning autonomously from circulatory support, and the surgeon may visually observe acute distension of the left ventricle due to the failure coaptation of the aortic valve leaflets. Several case reports^8,11,12,15,17,19,20^ underscore the importance of transesophageal echocardiography (TEE) during surgery, upon weaning from the CPB machine, to assess the favorable result of the intervention. However, acute AR may sometimes not be readily apparent, remaining clinically unrecognized. Serial echocardiogram examinations and clinical assessments should be conducted to detect any delayed onset of AR.^10,11^ In addition to its role in monitoring during surgery, echocardiography is crucial in the immediate post-operative period, particularly in the intensive care unit (ICU). Timely assessment of cardiac function using both transthoracic (TTE) and TEE is essential for detecting immediate complications following cardiac surgery. For example, rapid TTE in the ICU, often performed before extubating the patient, is vital for promptly evaluating hemodynamic status and cardiac function, and identifying any remaining valvular dysfunction.^24,25^

 Furthermore, as patients move towards discharge, echocardiography remains integral to their care.^25^ This pre-discharge TTE assessment is a key aspect of the multidisciplinary approach to post-operative care, aiding in the identification of any ongoing issues that may require further attention or intervention before the patient moves to outpatient management.

 While TEE stands as the gold standard diagnostic test, the efficacy of mid-esophageal views is often compromised due to ultrasound artifacts stemming from the prosthetic valves. The incorporation of three-dimensional (3D) TEE emerges as a pivotal enhancement, providing a more detailed imaging approach that makes the diagnosis of valve perforation easier.^10,26^

###  Management

**Figure 2:**
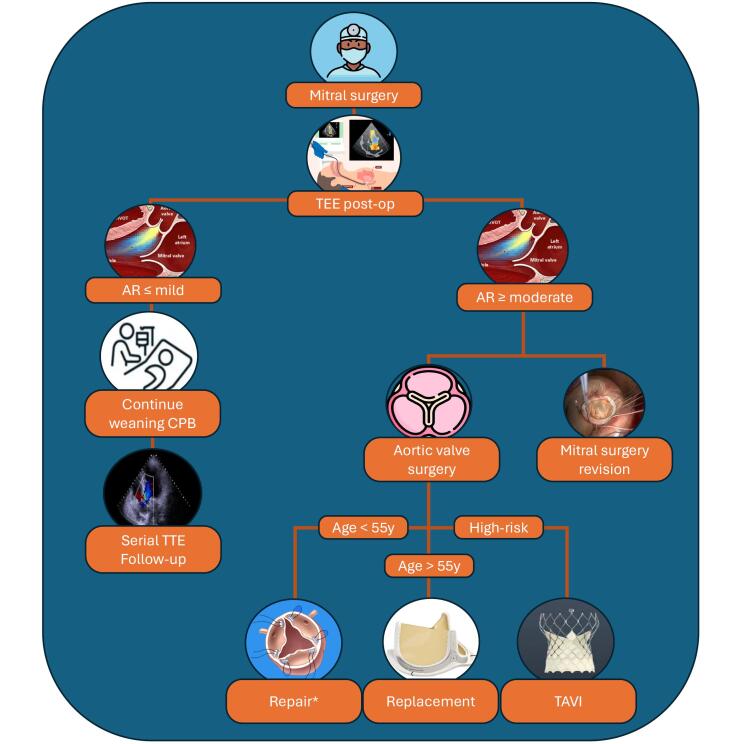
Suggested flowchart of iatrogenic aortic insufficiency after mitral valve surgery *Aortic valve repair may be considered in selected patients at experienced centers. TEE: trans-esophageal echocardiography; TTE: trans-thoracic echocardiography. AR: Aortic regurgitation; CPB: cardiopulmonary bypass; post-op: post-operative.

 Iatrogenic valvular regurgitation, arising as a consequence of leaflet entrapment or perforation, has been reported following a spectrum of cardiac procedures,^27^ especially after mitral valve surgery. Management of acute AR following mitral valve surgery is a multifaceted challenge that requires a meticulous and timely approach. Immediate recognition of the AR is paramount, and if visualized during the weaning from the CPB machine, the surgical team must act promptly. While mild AR can be medically managed, ≥ moderate regurgitation arising from perforated cusps mandates repair through the application of a pericardial patch or aortic valve replacement, contingent upon the extent of the damage. Moreover, it is essential to emphasize the significance of collegial discussion within the operating room, involving the surgeon, anesthesiologist, and cardiologist, after aortic declamping but before CPB weaning. This collaborative approach facilitates the determination of the optimal strategy and underscores its importance for patient follow-up, particularly if a decision is made to pursue a conservative management approach and monitor the patient through serial echocardiogram examinations. The strategies adopted to address this issue are diverse and heterogeneous, ranging from medical therapy for mild AR to immediate or delayed surgical interventions for larger AR severity. We propose a possible decision algorithm in Figure 2. Notably, the emergence of transcatheter aortic valve implantation (TAVI) as a potential alternative approach in non-severe AR but with calcified leaflets is highlighted by Spina et al.^21^ TAVI remains an attractive option for patients at high surgical risk, but the deformation of the aortic annulus after mitral surgery and the higher risk of associated paravalvular leaks compared with surgery should not be neglected. In fact, a case report detailing an unfortunate outcome following cardiac transplantation as treatment strategy was presented by Lee and colleagues.^16^ The multitude and variable approaches that have been described in literature cannot but jeopardize the clinical outcomes of those patients.

 The present review acknowledges some limitations. First, the presented data is likely underestimated, as the incidence and mortality in cases of iatrogenic AR after mitral valve surgery are presumed to be higher. This is attributed to potential publication bias, because of underreporting, particularly in those cases yielding unfavorable outcomes. Finally, the study comprised a restricted sample size, often encompassing diverse patients across various clinical conditions, potentially failing to capture the full breadth of relevant data.

## Conclusion

 Iatrogenic aortic valve regurgitation after mitral valve surgery remains a bothersome complication. Intraoperative transesophageal echocardiography plays a crucial role for early diagnosis. Management strategies vary from medical therapy to surgical interventions, depending on AR severity and patient’s surgical risk. The reparative strategy requires a complex surgical procedure burdened by significant mortality. Care should be taken to avoid this complicated scenario.

## Competing Interests

 Julien Ternacle: Consultant for Abbott, Edwards Lifesciences, General Electric, and Philips Healthcare; Thomas Modine: Proctor for Abbott Laboratories and Medtronic Inc., Consultant for Edwards Lifesciences; Basel Ramlawi: Consultant/Advisory Board for Medtronic Inc., Boston Scientific Corporation, AtriCure Inc., Corcym Inc.

## Supplementary Files

Supplementary file 1 contains Tables S1-2 and Figure S1.
