# Supplementary material for: Iatrogenic aortic valve injury following mitral valve surgery: A systematic review
Source: J Cardiovasc Thorac Res. 2025 Sep 28;17(3):153–8. doi: 10.34172/jcvtr.025.33350 (PMC12620141; doi:10.34172/jcvtr.025.33350)
Supplement: Supplementary file 1 — contains Tables S1-2 and Figure S1. [file jcvtr-17-153-s001.pdf]

J Cardiovasc Thorac Res. 2025;17(3):153-S1
doi: 10.34172/jcvtr.025.33350
http://jcvtr.tbzmed.ac.ir

Figure S1 – Anatomical structures around the aortic valve

LC = left cusp; M = mitral valve; NCC = non-coronary cusp; P = pulmonary valve; RC = right cusp; T = tricuspid valve. White star = interatrial septum; Black star = left bundle

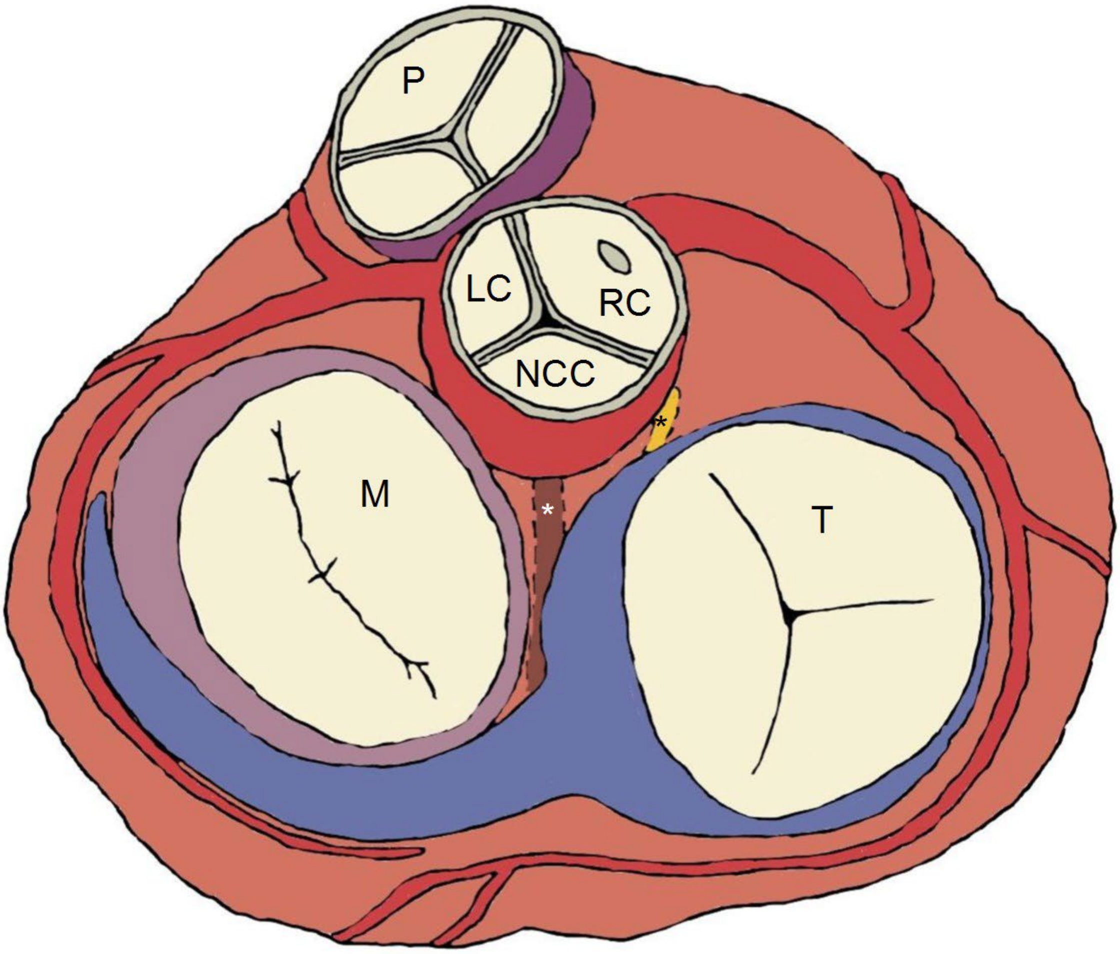

Supplementary Table 1 – Search strategy

#	Search
1	"aorta"[MeSH Terms] OR "aorta"[All Fields] OR "aortic"[All Fields] OR "aortics"[All Fields]
2	"leaflet"[All Fields] OR "leaflet's"[All Fields] OR "leaflets"[All Fields] OR "valve"[All Fields] OR "valve's"[All Fields] OR "valved"[All Fields] OR "valves"[All Fields] OR "valving"[All Fields] OR "cusp"[All Fields]
3	1 AND 2
4	"perforant"[All Fields] OR "perforants"[All Fields] OR "perforate"[All Fields] OR "perforated"[All Fields] OR "perforates"[All Fields] OR "perforating"[All Fields] OR "perforation"[All Fields] OR "perforations"[All Fields] OR "perforative"[All Fields] OR "perforator"[All Fields] OR "perforator's"[All Fields] OR "perforators"[All Fields]
5	"injurie"[All Fields] OR "injuried"[All Fields] OR "injuries"[Subheading] OR "injuries"[All Fields] OR "wounds and injuries"[MeSH Terms] OR ("wounds"[All Fields] AND "injuries"[All Fields]) OR "wounds and injuries"[All Fields] OR "injurious"[All Fields] OR "injury's"[All Fields] OR "injuryed"[All Fields] OR "injurys"[All Fields] OR "injury"[All Fields]

6	"iatrogen"[All Fields] OR "iatrogenic"[All Fields] OR "iatrogenically"[All Fields] OR "iatrogenicity"[All Fields] OR "iatrogenics"[All Fields] OR "iatrogenous"[All Fields]
7	3 AND (4 OR 5 OR 6)
8	"mitral valve"[MeSH Terms] OR ("mitral"[All Fields] AND "valve"[All Fields]) OR "mitral valve"[All Fields]
9	"surgery"[Subheading] OR "surgery"[All Fields] OR "surgical procedures, operative"[MeSH Terms] OR ("surgical"[All Fields] AND "procedures"[All Fields] AND "operative"[All Fields]) OR "operative surgical procedures"[All Fields] OR "general surgery"[MeSH Terms] OR ("general"[All Fields] AND "surgery"[All Fields]) OR "general surgery"[All Fields] OR "surgery's"[All Fields] OR "surgerys"[All Fields] OR "surgeries"[All Fields]
10	8 AND 9
11	7 AND 10

Supplementary Table 2 – Joanna Briggs Institute Critical Appraisal tool

Paper	Q1	Q2	Q3	Q4	Q5	Q6	Q7	Q8
Aboelnasr 2013 [6]	Yes	Yes	Yes	Yes	Yes	Yes	Yes	Yes
Al Yamani 2012 [7]	Yes	No	Yes	Yes	Yes	Yes	Yes	Yes
Alshoubi 2022 [8]	Yes	Yes	Yes	Yes	Yes	Yes	Yes	Yes
Dogan 2013 [9]	Yes	Yes	Yes	Yes	Yes	Yes	Yes	Yes
Dreyfus 2011 [10]	Yes	Yes	Yes	Yes	Yes	Yes	Yes	Yes
Ducharme 1999 [11]	Yes	Yes	Yes	Yes	Yes	No	Yes	Yes
Hill 1997 [12]	Yes	Yes	Yes	Yes	Yes	Yes	Yes	Yes
Koh 2002 [13]	Yes	Yes	Yes	Yes	Yes	No	No	Yes
Kolakalapudi 2015 [14]	Yes	Yes	Yes	Yes	Yes	No	No	Yes
Lakew 2016 [15]	Yes	No	Yes	Yes	Yes	No	No	Yes
Lee 1996 [16]	Yes	Yes	Yes	Yes	Yes	Yes	Yes	Yes
Mehta 2007 [17]	Yes	Yes	Yes	Yes	Yes	Yes	Yes	Yes
Oakley 2013 [18]	Yes	Yes	Yes	Yes	Yes	No	No	Yes
Rother 2000 [19]	Yes	No	Yes	Yes	Yes	No	No	No
Santiago 2011 [20]	Yes	Yes	Yes	Yes	Yes	No	No	Yes
Spina 2018 [21]	Yes	Yes	Yes	Yes	Yes	Yes	Yes	Yes

Uygyr 2018 [22]	Yes	Yes	Yes	Yes	Yes	No	No	Yes

Q1. Were patient's demographic characteristics clearly described? Q2. Was the patient's history clearly described and presented as a timeline? Q3. Was the current clinical condition of the patient on presentation clearly described? Q4. Were diagnostic tests or assessment methods and the results clearly described? Q5. Was the intervention(s) or treatment procedure(s) clearly described? Q6. Was the post-intervention clinical condition clearly described? Q7. Were adverse events (harms) or unanticipated events identified and described? Q8. Does the case report provide takeaway lessons?
